# Single Nucleotide Polymorphisms in *SLC5A1*, *CCNA1*, and *ABCC1* and the Association with Litter Size in Small-Tail Han Sheep

**DOI:** 10.3390/ani9070432

**Published:** 2019-07-09

**Authors:** Yongfu La, Qiuyue Liu, Liping Zhang, Mingxing Chu

**Affiliations:** 1 College of Animal Science and Technology, Gansu Agricultural University, Lanzhou 730070, China; 2 Key Laboratory of Animal Genetics and Breeding and Reproduction of Ministry of Agriculture, Institute of Animal Science, Chinese Academy of Agricultural Sciences, Beijing 100193, China

**Keywords:** *SLC5A1*, *CCNA1*, *ABCC1*, expression, sheep

## Abstract

**Simple Summary:**

Litter size is one of the most important reproductive traits in sheep. Four single nucleotide polymorphisms (SNPs), g.70067210 T > C in *SLC5A1*, g.25350431 C > T and g.25360220 T > C in *CCNA1*, and g.14413132 C > T in *ABCC1*, were identified by mass spectrometry and may be associated with litter size in sheep. Four SNPs were genotyped in Small-Tail Han, Hu, Cele Black, Suffolk, Sunite, Prairie Tibetan, and Tan sheep, and the expression patterns of *SLC5A1*, *CCNA1*, and *ABCC1* were determined in Small-Tail Han sheep with different fecundities. Furthermore, we also studied the *FecB* mutation’s association with litter size in Small-Tail Han sheep. The results indicated that all genes included in this study were differentially expressed in the ovary and uterus of polytocous and monotocous Small-Tail Han sheep. Furthermore, association analysis indicated that both g.70067210 T > C in *SLC5A1* and the *FecB* mutation in *BMPR-IB* were significantly associated with litter size in Small-Tail Han sheep. Linear regression analysis of the association of multiple markers (*FecB* and g.70067210 T > C in *SCL5A1*) with litter size indicated that homozygous ewes carrying the BB/TT genotype had a larger litter size than any other genotype.

**Abstract:**

*SLC5A1*, *CCNA1*, and *ABCC1* have been extensively studied as candidate genes because of their great influence on the reproductive traits of animals. However, little is known about the association between polymorphisms of the *SLC5A1*, *CCNA1*, and *ABCC1* genes and litter size in Small-Tail Han sheep. In this study, the expression levels of *SLC5A1*, *CCNA1*, and *ABCC1* in HPG (hypothalamic–pituitary–gonadal) axis tissues of polytocous and monotocous Small-Tail Han sheep were analyzed by qPCR. To better understand the effects of four single nucleotide polymorphisms (SNPs) comprising of g.70067210 T > C in *SLC5A1*, g.25350431 C > T and g.25360220 T > C in *CCNA1*, and g.14413132 C > T in *ABCC1*, a population genetic analysis was conducted using data obtained from genotyping in 728 sheep from seven breeds. The results indicated that all genes included in this study were differentially expressed in the pituitary and uterus of polytocous and monotocous Small-Tail Han sheep (*p* < 0.05). The associations of these four SNPs and the *FecB* mutation with litter size in 384 Small-Tail Han sheep were analyzed, therefore, and it was found that both g.70067210T > C and the *FecB* mutation were significantly associated with litter size (*p* < 0.05). The linear regression analysis of the association of multiple markers (*FecB* and g.70067210 T > C in *SCL5A1*) with litter size indicated that homozygous ewes carrying the BB/TT genotype had larger litter size than any ewes with any other genotype. In conclusion, the SLC5A1 SNPs significantly affect litter size in sheep and are useful as genetic marker for litter size.

## 1. Introduction

Sheep breeding, as an integral part of the Chinese animal industry, has economic benefits, which are affected by fertility and reproductive efficiency [1]. Although numerous different sheep breeds have been found, most breeds are monotocous, and only a few breeds have two or more lambs [2]. Therefore, improving reproductive efficiency is the focus of sheep breeding studies. Litter size is associated with low heritability and is one of the most important reproductive traits in sheep, controlled by genetics and the environment [3,4]. To date, the *FecB* mutation (c.746 A > G) in *BMPR-IB* (bone morphogenetic protein receptor IB) was identified as the major gene controlling prolificacy in sheep [5,6,7]. In addition, many genes associated with prolificacy were identified, including *BMP15* (bone morphogenetic protein 15) [8,9,10] and *GDF9* (growth differentiation factor 9) [11,12,13].

Solute carrier family 5 member 1 (*SLC5A1*), also known as *SGLT1*, is a member of the sodium-dependent glucose transporter (*SGLT*) family, allowing the transfer of glucose and galactose to intracellular sodium [14]. Glucose is the primary source of energy for metabolism and development in fetal and placental tissue [15]. Since the fetus is critically dependent on the net transfer of glucose across the placenta [16], during the final third of pregnancy in mammals, the fetal demand for glucose necessitates the presence of a rapid, high-volume system for maternal–fetal glucose transfer. This is regulated by the membrane-spanning glycoproteins family glucose transporter (*SLC2*) and the sodium-dependent glucose transporter family [17,18]. During pig pregnancy, large amounts of glucose and arginine are transported across the porcine placental membrane, a process that relies on *SLC5A1* to move glucose against its concentration gradient [19,20,21]. Studies have found that the porcine conceptuses begin to elongate and secrete E2, which increases the number of *SLC5A1* transporters in the uterine luminal epithelium, thereby increasing the transport of glucose into the uterine lumen, and improving the growth and development of the porcine conceptus [22]. *SLC5A1* has the same function in sheep. For example, in ewes, Arg and Gluc are significantly increased in the uterine lumen between days 10 and 15 of pregnancy, due to an increased expression of *SLC5A1* in uterine epithelia [23]. Pisani et al. found that *SLC5A1* plays an important role in the maturation of ovine oocytes [24]. Results of all these previous studies indicate that the *SLC5A1* gene has important functions in female reproduction. Whether the mutations of the *SLC5A1* gene affect sheep prolificacy, similar to what occurs as a result of mutations in the *BMPRIB* gene, remains to be elucidated.

Cyclin A1 (*CCNA1*) is an important cell-cycle regulator and a marker of late spermatocytes [25,26]. In animals, there are two types of cyclin A, *CCNA1* and *CCNA2*, both of which are expressed in male germ cells, suggesting that they play an important role in animal reproduction [27,28]. The disruption of mouse Ccna1 results in male infertility and complete spermatogenic arrest prior to the first meiotic division [29]. Similar to mouse *CCNA1*, human *CCNA1* is localized in late meiotic spermatocytes [30]. *CCNA1* plays an important regulatory role in the M phase of the oocyte meiotic cell cycle [31]. *ABCC1* (ATP-binding cassette subfamily C member 1) is a member of the ABC (ATP-binding cassette) superfamily, whose role as the primary transporter of many organic anions has been largely characterized [32]. Early studies have shown that *ABCC1* is directly involved in protecting fetal development from the deleterious consequences of xenobiotics [33,34]. Results of all these previous studies indicate that the *CCNA1* and *ABCC1* genes have important functions in female reproduction. However, few studies have investigated the effect of the *CCNA1* and *ABCC1* genes on litter size in sheep.

In the present study, the mRNA expression levels of *SLC5A1*, *CCNA1*, and *ABCC1* in HPG (hypothalamic–pituitary–gonadal) axis tissues of Small-Tail Han sheep were measured by qPCR. Then, based on previous data from the whole-genome sequencing (WGS) previously performed, four SNPs of the *SLC5A1*, *CCNA1*, and *ABCC1* genes were detected in 99 experimental sheep [35,36]. We explored the frequency of the four SNPs in seven sheep breeds and studied their association with litter size in Small-Tail Han sheep. Furthermore, we also studied the *FecB* mutation’s association with litter size in Small-Tail Han sheep. The purpose of this study was to investigate the effects of a novel gene involved in reproduction on the litter size of Small-Tail Han sheep and the effect of gene–gene combinations on litter size.

## 2. Materials and Methods

All the experimental procedures mentioned in the present study were approved by the Science Research Department (in charge of animal welfare issues) of the Institute of Animal Sciences, Chinese Academy of Agricultural Sciences (IAS-CAAS) (Beijing, China). Ethical approval on animal survival was given by the animal ethics committee of IAS-CAAS (No. IASCAAS-AE-03, 12 December 2016).

### 2.1. Animals

Based on a TaqMan assay using *FecB* mutant probes and three lambing records, three BB ewes (polytocous sheep, n = 3, average litter size ≥ 2) and three ++ ewes (monotocous sheep, n = 3, average litter size = 1) were selected from nucleus flocks of Small-Tail Han sheep in the southwest region of Shandong Province, China. The six selected sheep were healthy, each aged three years old, similar in weight, and fed in an indoor setting under similar conditions of room temperature, illumination, feeding system, and nutrition level. The six female sheep were slaughtered in autumn, when they accepted the teasing behavior for the advent of oestrum. The six female sheep were slaughtered; tissues from the HPG axis (hypothalamus, pituitary, ovary and uterus) were collected and immediately frozen in liquid nitrogen, then stored at −80 °C for RNA extraction.

Blood samples from 728 sheep of seven breeds were collected into EDTA-coated tubes and stored at −20 °C for DNA isolation. The seven sheep breeds in this study included Small-Tail Han (n = 384), Hu (n = 86), Cele Black (n = 71), Suffolk (n = 63), Sunite (n = 18), Prairie Tibetan (n = 82), and Tan sheep (n = 24).

### 2.2. Detection of SLC5A1, CCNA1, and ABCC1 Expression by Real-Time PCR

RNA was extracted using TRIzol reagent (TaKaRa, Dalian, China) according to the manufacturer’s instructions, and was treated with DNase using a TURBO DNA-free Kit (Ambion, Austin, TX, USA). The cDNA was generated by a PrimeScript^TM^ RT reagent kit (TaKaRa, Dalian, China).

*SLC5A1*, *CCNA1*, and *ABCC1* primers (exon-span) for real-time PCR were designed according to NM_001009404.1, XM_027973608.1, and XM_027961611.1, using Primer-BLAST (NCBI https://www.ncbi.nlm.nih.gov). Real-time PCR was performed at 95 °C for 10 min, followed by 95 °C for 15 s, 60 °C for 60 s for 45 cycles, and 72 °C for 30 s. qPCR was performed on the LightCycler 480II (Roche, Basel, Sweden) using the SYBR Green Real-time PCR Master Mix (TOYOBOCO, Ltd., Osaka, Japan). β-Actin was used as an internal reference to normalize target gene expression. All experiments were performed in triplicate. Table 1 lists the primers that were used for real-time PCR.

### 2.3. Genotyping

The g.70067210 T > C locus in the *SLC5A1* gene, g.25350431 C > T and g.25360220 T > C loci in the *CCNA1* gene, and the g.14413132 C > T locus in the *ABCC1* gene were selected for genotyping in 728 samples from Small-Tail Han, Hu, Cele Black, Suffolk, Sunite, Prairie Tibetan, and Tan sheep. The A > G locus in the *BMPR-IB* gene was selected for genotyping in 384 Small-Tail Han sheep. The single base extended primers used for detecting SNPs and polymerase chain reaction (PCR) programs were designed using MassARRAY Assay Design v.3.1, according to sheep *SLC5A1*, *CCNA1*, *ABCC1*, and *BMPR-IB* sequences available in the NCBI GenBank (Table 1). All sheep genomic DNA was subsequently genotyped using MassARRAY [37]. Genotyping was performed using PCR and primer extension and mass spectrometric analyses (iPlex assay, Sequenom, San Diego, CA, USA) on a Sequenom MassArray, according to the manufacturer’s instructions (http://www.sequenom.com). Polymerase chain reactions were carried out in 5 µL, containing 1.0 µL 20–50 ng/µL genomic DNA, 0.5 µL 10 × PCR buffer, 0.4 µL 25 mmol/L MgCl_2_, 0.1 µL 25 µmol/L dNTP, 1.0 µL PCR Primer mix, 0.2 µL Taq DNA polymerase (Promega, Madison, WI, USA), and ddH_2_O. PCR conditions were as follows: initial denaturation at 95 °C for 2 min, followed by 45 cycles of denaturation at 95 °C for 30 s, annealing at 56 °C for 30 s, extension at 72 °C for 60 s, with a final extension at 72 °C for 10 min. Primer extension reactions were carried out in 2 µL containing 0.2 µL iplex Buffer, 0.2 µL Terminator mix, 0.94 µL Extend primer mix, 0.04 µL iplex Enzyme, and ddH2O. Extension conditions were as follows: initial denaturation at 94 °C for 30 s, followed by 40 cycles of denaturation at 94 °C for 5 s, annealing at 52 °C for 5 s, with a final extension at 72 °C for 3 min. Only those samples with a >95% success rate and only those SNPs with a genotype success rate of >95% were included in the analysis.

### 2.4. Statistical Analysis

Allele frequencies, genotype frequencies, *p* values, polymorphism information content (*PIC*), heterozygosity (*HE*), and number of effective alleles (*NE*) were calculated using the data obtained from genotyping results. Sheep populations with *p* > 0.05 (chi-square test) were considered to conform to the Hardy–Weinberg equilibrium [38]. Tag mutations were selected using the general linear model (GLM) program of SPSS 16.0 for Windows (SPSS, Chicago, IL, USA) to analyze the association between single markers and litter size, defined as follows:

*y_ijn_* = *μ* + *P_i_* + *G_j_* + *I_PG_* + *e_ijn_*.

In this model, *y_ijn_* represents phenotypic value (litter size); *μ* is the population mean; *P_i_* shows the fixed effect of the *i*th parity (*i* = 1, 2, or 3); *G_j_* represents the effect of the *j*th genotypes (*j* = 1, 2, or 3); *I_PG_* represents the interactive effect of parity and genotype; and *e_ijn_* represents random error. All statistically significant single marker-traits associated with average litter size were initially included in further multiple-marker analysis of the combined effects of different genes on average litter size. The QTMs (quantitative trait modes) model is LS_Aa_ ≈ (LS_AA_ + LS_aa_)/2, where LS_Aa_ is the least square means of heterozygous individuals, LS_AA_ is the least square means of homozygous individuals with higher litter size performance, and LS_aa_ is the least square means of homozygous individuals with lower litter size performance [39]. A linear regression analysis of QTM was performed using SPSS 16.0 for Windows (SPSS, Chicago, IL, USA). *p* < 0.05 was considered to indicate statistical significance.

## 3. Results

### 3.1. Expression of SLC5A1, CCNA1, and ABCC1 in the HPG Axis of Small-Tail Han Sheep with Different Litter Sizes

**Figure 1:**
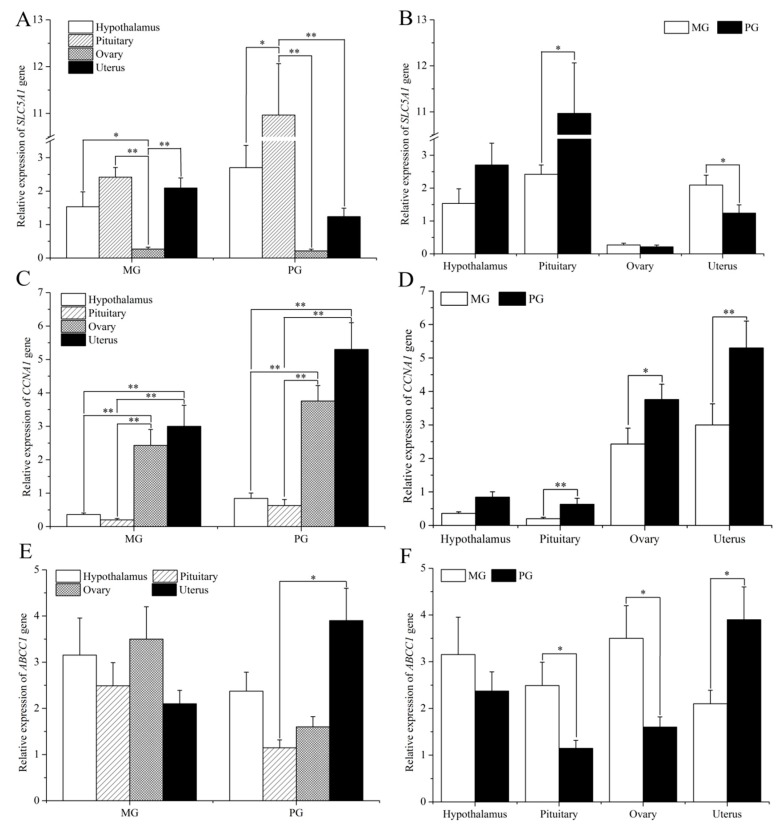
Expression of *SLC5A1*, *CCNA1*, and *ABCC1* in reproductive tissues of Small-Tail Han sheep. (**A**) Expression of *SLC5A1* in the hypothalamic–pituitary–gonadal (HPG) axis of Small Tailed Han sheep; (**B**) expression of *SLC5A1* in reproductive tissues of polytocous and monotocous Small-Tail Han sheep; (**C**) expression of *CNNA1* in the HPG axis of Small-Tail Han sheep; (**D**) expression of *CNNA1* in reproductive tissues of polytocous and monotocous Small-Tail Han sheep; (**E**) expression of *ABCC1* in the HPG axis of Small-Tail Han sheep; (**F**) expression of *ABCC1* in reproductive tissues of polytocous and monotocous Small-Tail Han sheep. Note: *: *p* < 0.05; **: *p* < 0.01.

The expression levels of *SLC5A1*, *CCNA1*, and *ABCC1* in the HPG axis tissues of polytocous and monotocous Small Tailed Han sheep were measured by qPCR. As shown in Figure 1, *SLC5A1* was expressed in four tissues of polytocous and monotocous Small-Tail Han sheep, with the highest level in the pituitary, the lowest level in the ovary, with no significant difference between the hypothalamus and uterus. The expression of *SLC5A1* in the hypothalamus and pituitary of polytocous Small-Tail Han sheep was higher than that in monotocous Small-Tail Han sheep, but in the uterus, expression was lower in polytocous Small-Tail Han sheep compared with that in monotocous Small-Tail Han sheep. However, except for the pituitary and uterus (*p* < 0.05), the expression of *SLC5A1* was not significantly different between the polytocous and monotocous Small-Tail Han sheep (*p* > 0.05).

*CCNA1* was expressed in four tissues of Small-Tail Han sheep, with the highest level in the uterus (*p* <0.01), followed by the ovary (*p* < 0.01), with no significant difference among the hypothalamus and pituitary, and uterus and ovary. The expression of *CCNA1* in the HPG tissues of polytocous Small-Tail Han sheep was higher than that in monotocous Small-Tail Han sheep. Except for the hypothalamus, the expression of the *CNNA1* gene was significantly different in the pituitary (*p* < 0.01), ovary (*p* < 0.05) and uterus (*p* < 0.01) of polytocous and monotocous Small-Tail Han sheep. The *ABCC1* was expressed in four tissues of Small-Tail Han sheep, and there were no significant differences among the tissues. The expression of *ABCC1* in the hypothalamus, pituitary and ovary of polytocous Small-Tail Han sheep was significantly lower than that in monotocous Small-Tail Han sheep, but in the uterus, expression was significantly higher in polytocous Small-Tail Han sheep compared with that in monotocous Small-Tail Han sheep (*p* < 0.05).

### 3.2. Population Genetic Analysis of Polymorphism in the SLC5A1, CCNA1 and ABCC1 Genes

Population genetic analyses of the g.70067210 T > C locus of the *SLC5A1* gene in seven sheep breeds were performed. The results are shown in Table 2. The results show that the T allele was more frequent than the C allele, with the TT genotype predominating in all populations. The g.70067210 T > C locus was moderately polymorphic (0.25 < *PIC* < 0.5) in Small-Tail Han, Suffolk, Hu, and Cele black sheep, with a low rate of polymorphism (*PIC* < 0.25) in Tan, Sunite, and Prairie Tibetan sheep. The chi-square test indicated that the Tan, Sunite, and Prairie Tibetan sheep were under Hardy–Weinberg equilibrium (*p* > 0.05), while the Small-Tail Han, Hu, Suffolk, and Cele black sheep were not.

Population genetic analyses of the g.25350431 C > T and g.25360220 T > C loci of the *CCNA1* gene in seven sheep breeds were performed (Table 2). The results show that the g.25350431 C > T locus T allele was more frequent than the C allele, with the TT genotype predominating in all populations. The g.25350431 C > T locus was moderately polymorphic (0.25 < *PIC* < 0.5) in Sunite and Prairie Tibetan sheep, with a low rate of polymorphism (*PIC* < 0.25) in other sheep breeds. The results show that the g.25360220 T > C locus C allele was more frequent than the T allele, with the CC genotype predominating in all populations. In addition to Prairie Tibetan sheep, the g.25360220 T > C locus was moderately polymorphic (0.25 < *PIC* < 0.5) in other sheep breeds. The chi-square test for the g.25350431 C > T and g.25360220 T > C loci indicated that all sheep breeds were under the Hardy–Weinberg equilibrium (*p* > 0.05).

Population genetic analyses of the g.14413132 C > T locus of the *ABCC1* gene in seven sheep breeds were performed (Table 2). The results showed that the g.14413132 C > T locus C allele was more frequent than the T allele, with the CC genotype predominating in all populations. The g.25350431 C > T locus was moderately polymorphic (0.25 < *PIC* < 0.5) in Small-Tail Han, Hu, Sunite, and Cele black sheep, with a low rate of polymorphism (*PIC* < 0.25) in the Tan, Suffolk, and Prairie Tibetan breeds. The chi-square test indicated that all sheep breeds except Small-Tail Han sheep were under the Hardy–Weinberg equilibrium (*p* > 0.05).

### 3.3. Association Analysis of SNPs with Litter Size in Small-Tail Han Sheep

To better understand the association of the g.70067210 T > C locus in the *SLC5A1* gene, the g.25350431 C > T and g.25360220 T > C loci in the *CCNA1* gene, the g.14413132 C > T locus in the *ABCC1* gene, and the *FecB* mutation with litter size, an association analysis was performed for the SNPs in terms of litter size in 384 Small-Tail Han sheep. The least squares means and standard errors for litter size are shown in Table 3. In the g.70067210 T > C site, the mean litter sizes of the first, second, and third parities for TT and TC were significantly higher those of the CC genotype (*p* < 0.05). However, a comparison of litter size between TT and TC showed no significant difference (*p* > 0.05). Furthermore, a comparison of average litter sizes among TT, TC, and CC showed a significant difference (*p* < 0.05). The g.25350431 C > T, g.25360220 T > C and g.14413132 C > T sites were not significantly associated with any of the three parities of litter size (*p* > 0.05). The *FecB* mutation was significantly associated with litter size at all three parities, and sheep with the BB variant had the largest litters (*p* < 0.05).

### 3.4. Regression Analysis of SLC5A1 and BMPR-IB Associated with Average Litter Size in Small-Tail Han Sheep

**Figure 2:**
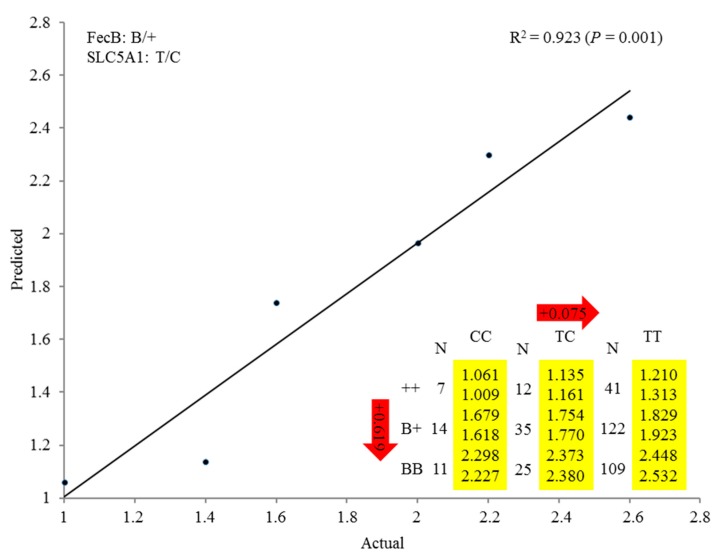
Genetic networks associated with average litter size in Small-Tail Han sheep. The numbers in arrows represent the substitution effects of one genotype or allele on another. Predicted (top) and actual (bottom) performance values for combined genotype(s).

The linear regression analysis of SNPs and their QTMs associated with each single trait confirmed the *SLC5A1* and *BMPR-IB* gene associations with litter size and revealed the *SLC5A1/BMPR-IB* gene network (Figure 2). *SLC5A1/BMPR-IB* was identified as an additive–additive combination in terms of the role of gene combinations in the two-gene network. This linear regression analysis also revealed a high correlation between predicted and actual performance values for the combined genotypes (0.923, *p* < 0.05). Among the nine genotypes of the *SLC5A1/BMPR-IB* network, the TT/BB and CC/++ genotypes were associated with the maximum and minimum average litter sizes, respectively.

## 4. Discussion

The tissue expression pattern of *SLC5A1* has been examined in many animals, with high expression levels in the small intestine [40], ovary [41], and uterus [22]. Human *CCNA1* is expressed most highly in the testis and, at lower levels, in the adult brain and in hematopoietic cells [42]. In mammalians, *CCNA1* appears to be stage-specific and highly expressed in germ cells [31,43]. *ABCC1* is widely expressed in various tissues and plays an important role in uteroplacental transport signaling [44,45]. In addition, it was reported that the hypothalamic–pituitary–gonadal axis (HPG) is the most important system for controlling mammalian reproduction [46]. In this study, *SLC5A1*, *CCNA1*, and *ABCC1* were expressed in four tissues of Small-Tail Han sheep. It was found that the expression levels of *SLC5A1* in the hypothalamus and pituitary of polytocous Small-Tail Han sheep were higher than that in monotocous Small-Tail Han sheep, indicating that the expression of *SLC5A1* can regulate the function of the hypothalamus and pituitary, which are especially important for mammalian reproduction. The expression level of *CCNA1* in the HPG of polytocous Small-Tail Han sheep was higher than that in monotocous Small-Tail Han sheep, indicating that the expression of *CCNA1* can positively regulate the function of the hypothalamus, pituitary, uterus, and ovary, thus affecting mammalian reproduction. The expression level of *ABCC1* in the uterus of polytocous Small-Tail Han sheep was higher than that in monotocous Small-Tail Han sheep, indicating that *ABCC1* may be associated with the prolificacy of Small-Tail Han sheep. These results preliminarily confirmed that *SLC5A1*, *CCNA1*, and *ABCC1* were closely associated with litter size in Small-Tail Han sheep.

In previous studies, *BMPR-IB* (the *FecB* mutation) was identified as the major gene affecting prolificacy in sheep [5,9]. According to previous reports, the *FecB* mutation was shown to be significantly associated with litter size in Small-Tail Han sheep [47,48,49], which is similar to the results of this study. Although it is reported that Small-Tail Han sheep carry a major mutation (*FecB*) for litter size [47,50], the existence of other major mutations related to litter size in this breed remains to be determined. However, the identification of other major or candidate genes associated with litter size is also important for further improving this characteristic in Small-Tail Han sheep. The *SLC5A1* gene plays an important role in animal reproduction [51,52]. At present, only in humans has it been found that the *SLC5A1* gene mutation can cause glucose and galactose malabsorption [53,54]. Steinhauser et al. reported *SLC5A1* as an excellent candidate gene for placental transport of glucose during pregnancy to support the growth and development of the porcine conceptus [51]. Dorniak et al. also reported that *SLC5A1* regulates sheep endometrial function, which is important for conceptual growth and development during gestational implantation [52]. *CCNA1* is an important cell cycle regulator that plays an important regulatory role in germ cell meiosis, indicating that it plays an important role in animal reproduction [29,30,31,55]. *ABCC1* is associated with the transport of several compounds or ions across biomembranes and is crucial for successful fertilization [56]. However, few studies have investigated the effect of the *SLC5A1*, *CCNA1*, and *ABCC1* genes on litter size in sheep.

In the present study, four SNPs from the candidate genes *SLC5A1*, *CCNA1*, and *ABCC1* were selected for an analysis of the effects of single and multiple-marker combinations on litter size in Small-Tail Han sheep. The present study indicates that g.70067210 T > C, g.25360220 T > C, and g.25350431 C > T have a strong potential for selection in Small-Tail Han, Suffolk, Hu, and Cele black sheep, and that g.25350431 C > T has a strong potential for selection in Sunite and Prairie Tibetan sheep. Therefore, we conclude that the selection intensity of loci in different sheep breeds is different. Association analysis showed that g.25350431 C > T, g.25360220 T > C, and g.14413132 C > T mutations had no effect on the litter size of Small-Tail Han sheep, while the g.70067210 T > C mutation had a great effect on litter size in Small-Tail Han sheep. This suggests that the g.70067210 T > C mutation has potential functional significance in Small-Tail Han sheep reproduction, although its underlying mechanism remains to be elucidated.

Several studies using GLM for analysis have demonstrated the effect of multiple marker combinations on litter size in sheep, such as *BMPRIB/BMP-15* [5,48]. In this study, the significant markers along with their QTMs were integrated into a linear regression model to explore the effect of combined genotypes on litter size. This analysis revealed a high correlation between the predicted and actual values of litter size, suggesting that the linear regression model can also be used to assess the association between litter size and non-quantitative traits in sheep. In addition, linear regression analysis showed that sheep with the BB/TT genotype had larger litter sizes compared to sheep with only one predominant genotype. This indicates that litter size in Small-Tail Han sheep is influenced by multiple markers. Furthermore, our results suggest that homozygous individuals with the BB/TT genotype should remain in the MAS (marker-assisted selection) breeding program to increase the frequency of favorable alleles in the Small-Tail Han sheep population.

## 5. Conclusions

In this study, we found that the expression levels of *SLC5A*, *CCNA1*, and *ABCC1* may have a significant effect on litter size in Small-Tail Han sheep, and this preliminary result needs to be validated in further studies employing a large sample size. However, the g.70067210 T > C mutations had a significant effect on litter size for Small-Tail Han sheep, while the g.25350431 C > T, g.25360220 T > C, and g.14413132 C > T mutations did not. A linear regression analysis of the association of multiple markers with litter size indicated that homozygous ewes carrying the BB/TT genotype had a larger litter size than any other genotype; therefore, this genotype has important value for developing breeding programs to improve litter size in Small-Tail Han sheep.

## Figures and Tables

**Table 1:** Primer information.

Primer Name	Primer Sequence	Product Size	Usage
SLC5A1-F_1_	ACGTTGGATGTTTCAGGCTTAACCCGAGTG	98	PCR for g.70067210 T > C
SLC5A1-R_1_	ACGTTGGATGGGTAAAGCATATTCCCCCAG		
SLC5A1-E	TCCCCCAGAACCATTGCCTTG		Extension reaction
CCNA1-F_1_	ACGTTGGATGGCTTCTCTAAGGTACTGATG	112	PCR for g.25360220 T > C
CCNA1-R_1_	ACGTTGGATGCACTCCCAGTCTGAAGATGC		
CCNA1-E_1_	ACTGATGAATTTCTTCAGCATA		Extension reaction
CCNA1-F_2_	ACGTTGGATGCTAGTTTGACACATCATCTC	104	PCR for g.25350431 C > T
CCNA1-R_2_	ACGTTGGATGACAATTCAGTGTGGCGGTTC		
CCNA1-E_2_	TTTGACACATCATCTCAGTGTTA		Extension reaction
ABCC1-F_1_	ACGTTGGATGCTAATGTGGCCGTGTCTCTG	125	PCR for g.14413132 C > T
ABCC1-R1	ACGTTGGATGTGCCGCCACTCATGACTATG		
ABCC1-E	CACGCATGGCATCAG		Extension reaction
BMPR-IB-F	GTCGCTATGGGGAAGTTTGGATG	140	TaqMan for A746G
BMPR-IB-R	CAAGATGTTTTCATGCCTCATCAACACGGTC		
SLC5A1-F_2_	TCAGCACAAAGTGGACAGCTC	192	qPCR
SLC5A1-R_2_	CCCGGTTCCATAGGCAAACTCG		
CCNA1-F_3_	ACAGTTTCCCCTATGCTGGT	164	qPCR
CCNA1-R_3_	TTCCTCATGTAGTGTGCCTT		
ABCC1-F_2_	ATGACGCATCTCAACAAAGCC	101	qPCR
ABCC1-R_2_	TTGCCCATACTTCTTTCCCAG		
β-Actin-F	CCAACCGTGAGAAGATGACC	97	qPCR
β-Actin-R	CCCGAGGCGTACAGGGACAG		

**Table 2:** Population genetic analysis of all loci in seven sheep breeds.

Gene Name	Locus	Breed	Genotype Frequency			Allele Frequency		*PIC*	*HE*	*NE*	Chi-Square Test (*p*-Value)
*SLC5A1*	g.70067210T > C		TT	TC	CC	T	C				
		Small-Tail Han sheep	0.72(275)	0.20(75)	0.08(34)	0.81	0.19	0.26	0.30	1.43	0.00
		Tan sheep	0.71(17)	0.29(7)	0.00(0)	0.85	0.15	0.22	0.25	1.33	0.70
		Sunite sheep	0.78 (14)	0.22(4)	0.00(0)	0.89	0.11	0.18	0.20	1.25	0.87
		Suffolk sheep	0.73(46)	0.16(10)	0.11(7)	0.81	0.19	0.26	0.31	1.45	0.00
		Hu sheep	0.72(62)	0.21(18)	0.07(6)	0.83	0.17	0.25	0.29	1.40	0.04
		Cele black sheep	0.64(46)	0.23(16)	0.13(9)	0.76	0.24	0.30	0.36	1.57	0.00
		Prairie Tibetan sheep	0.70(58)	0.26(21)	0.04(3)	0.84	0.16	0.24	0.28	1.38	0.82
*CCNA1*	g.25350431 C > T		CC	CT	TT	C	T				
		Small-Tail Han sheep	0.02(7)	0.24(91)	0.74(277)	0.14	0.86	0.21	0.24	1.32	0.99
		Tan sheep	0.05(1)	0.19(4)	0.76(16)	0.14	0.86	0.21	0.24	1.32	0.60
		Sunite sheep	0.14(3)	0.24(5)	0.62(13)	0.26	0.74	0.31	0.39	1.63	0.21
		Suffolk sheep	0.00(0)	0.13(8)	0.87(52)	0.07	0.93	0.12	0.12	1.14	0.86
		Hu sheep	0.04(3)	0.18 (15)	0.78(62)	0.13	0.87	0.20	0.23	1.30	0.28
		Cele black sheep	0.03(2)	0.22(14)	0.75(48)	0.14	0.86	0.21	0.24	1.32	0.75
		Prairie Tibetan sheep	0.05(4)	0.34 (27)	0.61(48)	0.22	0.78	0.29	0.34	1.53	0.99
	g.25360220 T > C		TT	CT	CC	T	C				
		Small-Tail Han sheep	0.06(23)	0.34(129)	0.60(232)	0.23	0.77	0.29	0.35	1.54	0.67
		Tan sheep	0.08(2)	0.40(10)	0.52(12)	0.29	0.71	0.32	0.41	1.70	0.99
		Sunite sheep	0.05(0)	0.53(10)	0.42(8)	0.28	0.72	0.32	0.40	1.67	0.26
		Suffolk sheep	0.14(9)	0.30(19)	0.56(35)	0.29	0.71	0.33	0.41	1.71	0.10
		Hu sheep	0.08(7)	0.33(28)	0.59(51)	0.24	0.76	0.30	0.37	1.59	0.55
		Cele black sheep	0.03(2)	0.38(27)	0.59(41)	0.22	0.78	0.29	0.34	1.53	0.61
		Prairie Tibetan sheep	0.01(1)	0.25(20)	0.74(61)	0.13	0.87	0.21	0.23	1.30	0.90
*ABCC1*	g.14413132 C > T		CC	CT	TT	C	T				
		Small-Tail Han sheep	0.71(270)	0.24(94)	0.05(20)	0.83	0.17	0.25	0.29	1.40	0.01
		Tan sheep	0.96(23)	0.04(1)	0.00(0)	0.98	0.02	0.04	0.04	1.04	0.99
		Sunite sheep	0.56(10)	0.44(8)	0.00(0)	0.78	0.22	0.29	0.35	1.53	0.48
		Suffolk sheep	0.76(48)	0.19(12)	0.05(3)	0.86	0.14	0.21	0.24	1.32	0.21
		Hu sheep	0.56(48)	0.34(29)	0.10(9)	0.73	0.27	0.32	0.40	1.66	0.38
		Cele black sheep	0.58(41)	0.35(25)	0.07(5)	0.75	0.25	0.30	0.37	1.59	0.91
		Prairie Tibetan sheep	0.76(62)	0.20(17)	0.04(3)	0.86	0.14	0.21	0.24	1.31	0.43

Note: *PIC*, *HE* and *NE* represent polymorphic information content, heterozygosity and effective number of alleles, respectively; numbers in the parentheses represent the number of detected sheep of each genotype; *p* > 0.05 indicates the locus was under Hardy-Weinberg equilibrium.

**Table 3:** Associations between the genotypes of the single nucleotide polymorphisms (SNPs) and litter size in Small-Tail Han sheep.

Gene Name	Locus	Genotype	Litter Size (Means ± S.E.)			
			First Parity (N)	Second Parity (N)	Third Parity (N)	Average (N)
*SLC5A1*	g.70067210 T > C	TT	1.93 ± 0.04 ^a^ (275)	2.19 ± 0.05 ^a^ (165)	2.59 ± 0.11 ^a^ (64)	2.29 ± 0.07 ^a^ (275)
		TC	1.92 ± 0.08 ^a^ (75)	2.16 ± 0.12 ^a^ (44)	2.52 ± 0.21 ^a^ (18)	1.96 ± 0.10 ^b^ (75)
		CC	1.26 ± 0.12 ^b^ (34)	1.50 ± 0.17 ^b^ (21)	1.70 ± 0.28 ^b^ (10)	1.34 ± 0.11 ^c^ (34)
*CCNA1*	g.25360220 C > T	TT	1.89 ± 0.05 (23)	2.27 ± 0.08 (9)	2.00 ± 0.05 (4)	1.96 ± 0.13 (23)
		CT	1.77 ± 0.07 (129)	1.93 ± 0.04 (89)	2.28 ± 0.24 (25)	1.87 ± 0.14 (129)
		CC	1.90 ± 0.07 (232)	2.17 ± 0.14 (160)	2.56 ± 0.19 (59)	1.98 ± 0.25 (232)
	g.25350431 C > T	TT	2.00 ± 0.06 (7)	2.25 ± 0.12 (5)	2.50 ± 0.16 (3)	2.17 ± 0.07 (7)
		CT	1.83 ± 0.06 (91)	2.06 ± 0.09 (50)	2.46 ± 0.18 (24)	1.93 ± 0.27 (91)
		CC	1.88 ± 0.08 (277)	2.12 ± 0.15 (169)	2.46 ± 0.26 (63)	1.96 ± 0.16 (277)
*ABCC1*	g.14413132C > T	TT	1.95 ± 0.12 (20)	2.20 ± 0.29 (10)	2.33 ± 0.08 (3)	2.01 ± 0.19 (20)
		CT	1.88 ± 0.14 (94)	2.17 ± 0.16 (53)	2.52 ± 0.20 (22)	1.99 ± 0.13 (94)
		CC	1.86 ± 0.08 (270)	2.09 ± 0.16 (166)	2.40 ± 0.15 (67)	1.94 ± 0.25 (270)
*BMPR-IB*	c.746 A > G	BB	2.20 ± 0.05 ^a^ (147)	2.53 ± 0.07 ^a^ (86)	2.82 ± 0.12 ^a^ (39)	2.33 ± 0.04 ^a^ (147)
		B+	1.88 ± 0.04 ^b^ (177)	2.07 ± 0.06 ^b^ (110)	2.35 ± 0.11 ^b^ (43)	1.95 ± 0.04 ^b^ (177)
		++	1.03 ± 0.07 ^c^ (60)	1.15 ± 0.11 ^c^ (33)	1.33 ± 0.24 ^c^ (9)	1.08 ± 0.06 ^c^ (60)

Note: Numbers in the parentheses next to litter size represent the number of sheep of each genotype; Different lower-case letters in the same group indicate significant difference (*p* < 0.05).
